# Food Preservatives

**Published:** 1899-11-25

**Authors:** 


### FOOD PRESERVATIVES.

The reports of the proceedings of the Departmental Committee appointed to inquire into the use of preservatives and colouring matter in food are distinctly unpleasant reading, showing, as they do, the unblushing manner in which preservatives are mixed with all kinds of food of a perishable nature. The time seems to have gone by when these things were done *sub rosâ*, and no hesitation is felt in stating openly that such things, especially, as borax and boracic acid are used as an ordinary routine in many branches of the provision trade. Even the butter which is brought unsalted in cold chambers from New Zealand and Australia is stated to contain boracic acid, and it was maintained by one witness that nothing would do the butter business more mischief than to stop the use of a "moderate" use of preservative. Entire prohibition, it was added, would exclude at least 30 to 40 per cent. of the "fine fresh butter" that now comes into the country, and would raise the price of butter by 3d. or 4d. a pound. On the other hand Mr. James Riley, representing the Liverpool Chamber of Commerce, said that if butter were made on the latest scientific principles there was not the slightest necessity for any preservative matter. In Denmark preservatives were, he said, forbidden, and his experience had been that Danish butter kept as well as, if not better than, any other class; and this, we take it, about expresses the truth of the matter. Good stuff, well made, will keep fairly well without preservatives, but by the aid of preservatives it is possible to put upon the market, in competition with it, stuff which, without, perhaps, being bad, is at least not so well or so carefully made; and it is cheaper to add the preservative than to improve the process. In regard to bacon, it would appear that the case is rather different. The use of preservatives has enabled the American packers to put upon the market a class of mild-cured bacon which really could hardly be produced in quantity, at any rate so far off, without employing some such means of preventing decomposition, and the public has got to like it; in fact, according to one witness, "the public seemed to require mild-cured meats which could not be produced without boracic acid and borax," and the result of investigation into the matter had been to show that, while about 25 per cent. of American and Canadian meats come in salt, 75 per cent. are treated with borax. According to the returns of the packers the bacon was "boraxed about 1 per cent., and then in ordinary weather it would keep about a month." When, however, we are told that the use of borax is a "necessity" in the making of this class of bacon, it is worth noting the statement that, from a commercial standpoint, the Liverpool Provision Trade Association are not advocates of borax, which suggests that they know pretty well that the trade would not vanish even if borax were abolished. Indeed, it is largely a question of where and how far off the stuff comes from, for it was stated that the Canadian bacon, which is very mild, is intended to compete with the Danish bacon. As with the butter, the addition of preservatives widens the field from which provisions can be drawn, and so, of course, to a certain extent lowers the price. No doubt from the "price" point of view there is something to be said for preservatives; but if, after full consideration, it should be decided to allow their use, we may at least demand that the thing should be done honestly and openly, and that preserved stuff should be sold as such, marked and declared not to be the pure and unadulterated article. Mr. Leonard Boseley said that milk, cream, butter, and condensed milk were most frequently treated with borax, boracic acid, formalin, and very occasionally with salicylic acid, and also with nitrates and sulphites. He mentioned, however, that a well-known London dairy company had for the last six years and a half used no preservative in any article except in a certain kind of potted cream, and not even in this for the past four years, and that they were prepared to run their business without preservatives at all. Nevertheless, 50 per cent. of the dairymen in London used preservatives of some sort.

